# The Fetal Growth Restriction at Term Managed by Angiogenic Factors Versus Feto-Maternal Doppler (GRAFD) Trial to Avoid Adverse Perinatal Outcomes: Protocol for a Multicenter, Open-Label, Randomized Controlled Trial

**DOI:** 10.2196/37452

**Published:** 2022-10-11

**Authors:** Pablo Garcia-Manau, Manel Mendoza, Erika Bonacina, Raquel Martin-Alonso, Lourdes Martin, Ana Palacios, Maria Luisa Sanchez, Cristina Lesmes, Ivan Hurtado, Esther Perez, Albert Tubau, Patricia Ibañez, Marina Alcoz, Nuria Valiño, Elena Moreno, Carlota Borrero, Esperanza Garcia, Eva Lopez-Quesada, Sonia Diaz, Jose Roman Broullon, Mireia Teixidor, Carolina Chulilla, Maria M Gil, Monica Lopez, Amparo Candela-Hidalgo, Andrea Salinas-Amoros, Anna Moreno, Francesca Morra, Oscar Vaquerizo, Beatriz Soriano, Marta Fabre, Elena Gomez-Valencia, Ana Cuiña, Nicolas Alayon, Jose Antonio Sainz, Angels Vives, Esther Esteve, Vanesa Ocaña, Miguel Ángel López, Anna Maroto, Elena Carreras

**Affiliations:** 1 Maternal Fetal Medicine Unit, Department of Obstetrics Vall d’Hebron Barcelona Hospital Campus Universitat Autònoma de Barcelona Barcelona Spain; 2 Maternal Fetal Medicine Unit, Department of Obstetrics Hospital Universitario de Torrejón Madrid Spain; 3 School of Medicine Universidad Francisco de Vitoria Madrid Spain; 4 Maternal Fetal Medicine Unit, Department of Obstetrics Hospital Universitari de Tarragona Joan XXIII Universitat Rovira i Virgili Tarragona Spain; 5 Department of Obstetrics Alicante University General Hospital Miguel Hernandez University Alicante Spain; 6 Alicante Institute for Health and Biomedical Research Alicante Spain; 7 Maternal Fetal Medicine Unit, Department of Obstetrics Hospital Clínico Universitario Virgen de la Arrixaca Universidad de Murcia Murcia Spain; 8 Maternal Fetal Medicine Unit, Department of Obstetrics Parc Taulí Hospital Universitari Universitat Autònoma de Barcelona Sabadell Spain; 9 Maternal Fetal Medicine Unit, Department of Obstetrics Hospital Universitari Germans Trias i Pujol Universitat Autònoma de Barcelona Badalona Spain; 10 Maternal Fetal Medicine Unit, Department of Obstetrics Hospital Universitario de Cabueñes Universidad de Oviedo Gijón Spain; 11 Maternal Fetal Medicine Unit, Department of Obstetrics Hospital Universitari Son Llàtzer Universitat de les Illes Balears Palma de Mallorca Spain; 12 Aragon Institute for Health Research Department of Obstetrics Hospital Clínico Universitario Lozano Blesa Zaragoza Spain; 13 Maternal Fetal Medicine Unit, Department of Obstetrics Fundació Althaia Universitat de Vic Manresa Spain; 14 Maternal Fetal Medicine Unit, Department of Obstetrics Hospital Universitario de A Coruña Universidade da Coruña A Coruña Spain; 15 Maternal Fetal Medicine Unit, Department of Obstetrics Hospital General Universitario de Elche Universidad Miguel Hernández Elche Spain; 16 Maternal Fetal Medicine Unit, Department of Obstetrics Hospital Universitario Virgen de Valme Universidad de Sevilla Sevilla Spain; 17 Maternal Fetal Medicine Unit, Department of Obstetrics Consorci Sanitari de Terrassa Universitat Internacional de Catalunya Terrassa Spain; 18 Maternal Fetal Medicine Unit, Department of Obstetrics Hospital Universitari Mútua Terrassa Universitat de Barcelona Terrassa Spain; 19 Maternal Fetal Medicine Unit, Department of Obstetrics Hospital Universitario de Getafe Universidad Europea de Madrid Getafe Spain; 20 Maternal Fetal Medicine Unit, Department of Obstetrics Hospital Universitario Puerta del Mar Universidad de Cádiz Cádiz Spain; 21 Maternal Fetal Medicine Unit, Department of Obstetrics Hospital Universitari de Girona Doctor Josep Trueta Universitat de Girona Girona Spain; 22 Maternal Fetal Medicine Unit, Department of Obstetrics Hospital Universitario Nuestra Señora de Candelaria Universidad de La Laguna Santa Cruz de Tenerife Spain

**Keywords:** fetal growth restriction, small for gestational age, PlGF, sFlt-1, Doppler, angiogenic factors

## Abstract

**Background:**

Fetal smallness affects 10% of pregnancies. Small fetuses are at a higher risk of adverse outcomes. Their management using estimated fetal weight and feto-maternal Doppler has a high sensitivity for adverse outcomes; however, more than 60% of fetuses are electively delivered at 37 to 38 weeks. On the other hand, classification using angiogenic factors seems to have a lower false-positive rate. Here, we present a protocol for the Fetal Growth Restriction at Term Managed by Angiogenic Factors Versus Feto-Maternal Doppler (GRAFD) trial, which compares the use of angiogenic factors and Doppler to manage small fetuses at term.

**Objective:**

The primary objective is to demonstrate that classification based on angiogenic factors is not inferior to estimated fetal weight and Doppler at detecting fetuses at risk of adverse perinatal outcomes.

**Methods:**

This is a multicenter, open-label, randomized controlled trial conducted in 20 hospitals across Spain. A total of 1030 singleton pregnancies with an estimated fetal weight ≤10th percentile at 36+0 to 37+6 weeks+days will be recruited and randomly allocated to either the control or the intervention group. In the control group, standard Doppler-based management will be used. In the intervention group, cases with a soluble fms-like tyrosine kinase to placental growth factor ratio ≥38 will be classified as having fetal growth restriction; otherwise, they will be classified as being small for gestational age. In both arms, the fetal growth restriction group will be delivered at ≥37 weeks and the small for gestational age group at ≥40 weeks. We will assess differences between the groups by calculating the relative risk, the absolute difference between incidences, and their 95% CIs.

**Results:**

Recruitment for this study started on September 28, 2020. The study results are expected to be published in peer-reviewed journals and disseminated at international conferences in early 2023.

**Conclusions:**

The angiogenic factor–based protocol may reduce the number of pregnancies classified as having fetal growth restriction without worsening perinatal outcomes. Moreover, reducing the number of unnecessary labor inductions would reduce costs and the risks derived from possible iatrogenic complications. Additionally, fewer inductions would lower the rate of early-term neonates, thus improving neonatal outcomes and potentially reducing long-term infant morbidities.

**Trial Registration:**

ClinicalTrials.gov NCT04502823; https://clinicaltrials.gov/ct2/show/NCT04502823

**International Registered Report Identifier (IRRID):**

DERR1-10.2196/37452

## Introduction

### Background

Fetal smallness affects around 10% of pregnancies [1]. Small fetuses are at a higher risk of intrauterine death and adverse perinatal outcomes [2]. In order to prevent these adverse outcomes, identification and appropriate management of small fetuses are crucial [3,4]. Based on gestational age (<32 weeks of gestation versus ≥32 weeks of gestation) at the time of disease onset, 2 distinct patterns of severity are observed in small fetuses, with the more severe cases being those with onset early in pregnancy (<32 weeks of gestation) [5]. In these cases, management is mainly based on fetal Doppler and indications for delivery are quite consistent [2], generally resulting in preterm neonates. However, most cases are diagnosed at a later gestational age (≥32 weeks) and, in this particular context, there is no clear consensus on the appropriate interventions to prevent adverse perinatal outcomes [6-8].

Moreover, the severity of fetal smallness is usually classified into 2 categories: fetal growth restriction (FGR), which is defined as a fetus failing to reach its genetically predetermined growth potential, and small for gestational age (SGA), which is defined as a fetus being small but without an increased risk of adverse perinatal outcomes. SGA fetuses are commonly referred to as constitutionally small fetuses [1,8]. Several criteria based on Doppler studies, growth velocity, and biometric percentiles are available to discriminate between SGA and FGR fetuses [2,8,9]. One of the most widely used classifications, as well as the one used in most maternity wards in Spain, is the one proposed by Figueras and Gratacós [8]. This classification, based on estimated fetal weight (EFW) and feto-maternal Doppler, allows the identification of the subset of small fetuses at a greater risk of perinatal complications (ie, true FGR fetuses) and the subset of small fetuses with a risk of perinatal complications similar to that of a normally growing fetus (ie, constitutionally small or SGA fetuses). According to several guidelines, FGR fetuses may benefit from early-term elective delivery (at 37-38 weeks), while SGA fetuses require closer monitoring, but not elective delivery until full term (39-40 weeks). FGR/SGA classification based on Doppler and EFW percentiles has a high sensitivity for adverse perinatal outcomes; nevertheless, more than 60% of fetuses with an EFW below the 10th percentile are classified as FGR and, therefore, will be delivered at 37 to 38 weeks [8]. Neonates delivered at 37+0 to 38+6 weeks+days of gestation are considered early-term and have poorer neonatal outcomes than full-term neonates (≥39 weeks of gestation) [10-12]. For this reason, early-term elective delivery should be restricted to FGR fetuses at an actual risk for adverse outcomes.

### Placental Insufficiency and SGA/FGR

The precise pathophysiology of SGA/FGR is unknown, but placental insufficiency is a common finding [13,14]. Several studies have reported histopathological findings related to placental malperfusion in SGA and FGR pregnancies [15,16]. The severity of the underlying placental insufficiency can be assessed by Doppler of the feto-maternal circulation [15,17]. Some studies have also shown an association between placental findings consistent with maternal vascular malperfusion and angiogenic imbalance involving a decrease of placental growth factor (PlGF), a proangiogenic factor, and an increase in soluble fms-like tyrosine kinase-1 (sFlt-1), an antiangiogenic factor, resulting in an increased sFlt-1/PlGF ratio [18-20].

### Management of SGA/FGR Pregnancies: EFW

As is widely known, there is an inversely proportional relationship between EFW and the risk of adverse perinatal outcomes [21-23]. For this reason, in DIGITAT (Disproportionate Intrauterine Growth Intervention Trial At Term), the only clinical trial ever conducted to evaluate the role of early-term elective delivery in improving perinatal outcomes of small fetuses, the only inclusion criterion was an EFW below the 10th percentile [24]. In that study, fetuses with an EFW below the 10th percentile were randomized into two groups: (1) early-term induction of labor and (2) expectant management until the onset of spontaneous labor. Perinatal outcomes were compared between the groups, showing that systematic early-term labor induction in pregnancies with small fetuses did not improve perinatal outcomes. By contrast, there was a significant increase in the number of admissions to the neonatal intensive care unit (NICU) and intermediate care unit for early-term neonates (51.1%) as compared to full-term neonates (39.8%). Since no differences were found in the baseline characteristics of the groups at enrollment, it is fair to assume that this 11.3% difference in neonatal admissions was mainly due to differences in gestational age at delivery between the groups. For this reason, a Cochrane review in 2015 [7] concluded that there is no evidence suggesting that early-term elective delivery of small fetuses (based only on EFW) should be recommended to avoid adverse perinatal outcomes. It must be noted that in DIGITAT, other factors predictive of poor prognosis in small fetuses, such as the amount of amniotic fluid, feto-maternal Doppler, or biophysical profile score, were not taken into account. Therefore, it might be possible that with more accurate identification of small fetuses who are actually at a higher risk of perinatal complications (ie, those with FGR), early-term elective delivery would have been found to improve perinatal outcomes as compared to the expectant management group.

### Management of SGA: Feto-Maternal Doppler

In recent years, and after the publication of DIGITAT, several studies have evaluated the role of feto-maternal circulation assessment by Doppler ultrasound in small fetuses [25-28]. These studies have shown that Doppler assessment allows identifying the subset of small fetuses at a higher risk of adverse perinatal outcomes (ie, those with FGR). Historically, umbilical artery (UA) pulsatility index (PI) assessed with Doppler has been considered the standard parameter to identify FGR. However, a large proportion of small fetuses with normal UA PI (ie, <95th percentile) have poorer perinatal outcomes than normally growing fetuses [21,29,30]. Thus, UA PI alone cannot be used to discriminate SGA from FGR fetuses [1,29]. Further studies showed that other Doppler parameters might have a greater predictive ability for adverse outcomes in late-onset SGA and FGR: cerebroplacental ratio (CPR), middle cerebral artery (MCA) PI, and uterine artery (UtA) PI [1,26,31,32]. According to these studies, abnormal CPR (ie, <5th percentile), MCA PI (ie, <5th percentile), or UtA PI (ie, >95th percentile) may be able to identify small fetuses at a higher risk of adverse outcomes (ie, FGR). A study including these criteria showed that small fetuses with abnormal Doppler parameters accounted for 60% of all small fetuses, indicating that more than half of fetuses with an EFW below the 10th percentile would be classified as FGR and that according to our current protocol, early-term induction of labor would therefore be recommended [25]. In the earlier study, induction of labor was recommended at 37 weeks of gestation in FGR fetuses (small fetuses with an EFW below the 3rd percentile or with an EFW below the 10th percentile accompanied by the presence of any abnormal Doppler parameter), while for other pregnancies with an EFW below the 10th percentile (ie, SGA fetuses) induction of labor was recommended at 40 weeks. Following that protocol, 134 cases (26.3%) had an adverse outcome, including, nonexclusively, 46 cases of neonatal acidosis and 106 cases of emergency cesarean delivery due to nonreassuring cardiotocography (CTG). Neonatal acidosis in that study was defined as a UA pH below 7.15 and a base excess greater than –12 mEq/L.

### Management of SGA: Angiogenic Factors

To date, few studies have evaluated the usefulness of angiogenic factors (AFs) in the management of late-onset or term SGA/FGR pregnancies. These studies show that the higher the sFlt-1/PlGF ratio, the worse the prognosis for small fetuses and the greater the risk of developing preeclampsia (PE), which in turn worsens maternal prognosis [19,33-35]. Recently, a large observational study compared the identification of term (36+0 to 37+6 weeks+days) small fetuses (EFW below the 10th percentile) at a higher risk of adverse outcomes using the standard Doppler assessment versus a new approach based on AFs [36]. In that study, 521 fetuses were identified as small, of which 102 had abnormal AF values (sFlt-1/PlGF ratio ≥38), whereas 412 had abnormal Doppler parameters. Therefore, according to the Doppler-based protocol, 79.1% (412/521) of small fetuses would have been classified as FGR, whereas according to the new AF-based approach, only 19.6% (102/521) of small fetuses would have been classified as FGR. By contrast, both approaches had a similar negative predictive value for adverse perinatal outcomes (99.3% and 99%, respectively), indicating a good, similar prognosis for those pregnancies not classified as FGR regardless of the classification used. Therefore, classification based on AFs seems more accurate and may have a lower rate of false positives than the Doppler-based protocol for the identification of small fetuses at a higher risk of adverse outcomes.

### Early-term Delivery: Short-term and Long-term Consequences

It might seem that whether a delivery is early term (<39 weeks) or full term (≥39 weeks) is not very relevant in terms of postnatal prognosis. However, several studies have found increased immediate postnatal morbidity (such as admission to the NICU due to a need for respiratory support) [10] and poorer long-term outcomes, such as the development of diabetes, obesity, and respiratory morbidity, in infants born early term as compared to full term [11,12]. Thus, a reduction in the number of elective early-term deliveries due to FGR overdiagnosis would certainly lead to improved short-term and long-term postnatal outcomes, ultimately resulting in healthier infants and adults.

### Rationale for the Study

The most common protocols used worldwide for the management of late-onset SGA/FGR are based on Doppler assessment, which recommends elective delivery at 37 weeks (or even earlier) in FGR pregnancies [25,37,38]. According to a classification based on Doppler parameters and EFW percentiles, up to 79.1% of small fetuses would be classified as FGR. By contrast, when using the AF-based approach (sFlt-1/PlGF ≥38), only 19.6% of small fetuses would be classified as FGR [36]. Additionally, both approaches seem to have a similar ability to identify small fetuses at risk (ie, those with FGR), which may benefit from an earlier elective delivery. Therefore, the AF-based protocol may potentially reduce by up to 75.2% (from 79.1% to 19.6%) the number of pregnancies classified as FGR (in which labor would be induced at 37 weeks) without worsening perinatal outcomes. Moreover, reducing the number of unnecessary labor inductions would not only improve patients’ perception of medical attention, but also would reduce the costs and risks derived from possible iatrogenic complications, which in turn would reduce the rate of cesarean deliveries. Additionally, fewer inductions would lower the rate of early-term neonates, thus improving neonatal outcomes and potentially reducing long-term infant metabolic, endocrine, and respiratory morbidities.

The sFlt-1/PlGF ratio has been shown to accurately predict PE and associated complications several weeks before onset [39-42]. Therefore, a management protocol based on AFs may potentially reduce the rate of PE and other maternal complications associated with PE, such as placental abruption or eclampsia.

### Objectives

#### Primary Objective

To determine whether the classification of small fetuses as FGR or SGA based on AFs is not inferior to the standard clinical approach (based on EFW and Doppler percentiles) for the identification of fetuses at a higher risk of adverse perinatal outcomes (neonatal acidosis and cesarean section due to nonreassuring CTG).

#### Secondary Objectives

To determine whether (1) the lower false-positive rate using AFs instead of Doppler to identify small fetuses as FGR results in a reduced number of elective deliveries before 38, 39, and 40 weeks, (2) a lower rate of early-term elective deliveries results in a reduced number of deliveries (elective and spontaneous) before 38, 39, and 40 weeks, (3) a lower rate of early-term elective deliveries results in a reduced number of cesarean deliveries, (4) a lower rate of early-term elective deliveries results in a reduced number of neonatal admissions to the NICU and a lower rate of adverse perinatal outcomes, (5) the AF-based approach reduces PE incidence in pregnancies with small fetuses, and (6) the AF-based classification reduces the incidence of placental-related complications.

## Methods

### Study Setting

The study will be conducted in 20 hospitals across Spain with experience in managing term SGA/FGR pregnancies: Vall d’Hebron Barcelona Hospital Campus (Barcelona), Hospital Universitario de Torrejón (Torrejón de Ardoz), Hospital Universitari de Tarragona Joan XXIII (Tarragona), Hospital General Universitario de Alicante (Alicante), Hospital Clínico Universitario Virgen de la Arrixaca (Murcia), Parc Taulí Hospital Universitari (Sabadell), Hospital Universitari Germans Trias i Pujol (Badalona), Hospital Universitario de Cabueñes (Gijón), Hospital Universitari Son Llàtzer (Palma de Mallorca), Hospital Clínico Universitario Lozano Blesa (Zaragoza), Fundació Althaia (Manresa), Hospital Universitario de A Coruña (A Coruña), Hospital General Universitario de Elche (Elche), Hospital Universitario Virgen de Valme (Sevilla), Consorci Sanitari de Terrassa (Terrassa), Hospital Universitari Mútua Terrassa (Terrassa), Hospital Universitario de Getafe (Getafe), Hospital Universitario Puerta del Mar (Cádiz), Hospital Universitari de Girona Doctor Josep Trueta (Girona), and Hospital Universitario Nuestra Señora de Candelaria (Santa Cruz de Tenerife).

### Trial Design

This is a multicenter, open-label, randomized clinical trial. The study design adheres to the SPIRIT (Standard Protocol Items: Recommendations for Interventional Trials) quality standard criteria for randomized trials [43]. A pragmatic approach will be adopted in order to evaluate the effectiveness of the intervention in real-life, routine practice conditions. Therefore, each participating site will use the fetal growth charts, Doppler reference values, and methods for cervical ripening and labor induction usually applied in their clinical practice.

The clinical trial was entered in the ClinicalTrials.org registry on August 6, 2020 (NCT04502823).

### Inclusion Criteria

Inclusion criteria at the time of enrollment are as follows: (1) age at least 16 years, (2) singleton pregnancy, (3) ultrasonographic EFW ≤10th percentile between 36+0 and 37+6 weeks+days of gestation, (4) sFlt-1/PlGF ratio measured between 36+0 and 37+6 weeks+days of gestation, (5) randomization between 36+0 and 37+6 weeks+days of gestation, and (6) gestational age confirmed by fetal crown-rump length measurement during the first trimester scan (from 11+0 to 13+6 weeks+days of gestation) or by in vitro fertilization dates.

### Exclusion Criteria

Exclusion criteria at the time of enrollment are as follows: (1) major fetal malformations or genetic disorders, (2) fetal death, (3) absent or reversed end-diastolic flow in UA Doppler, (4) nonreassuring CTG, (5) preeclampsia, (6) diminished fetal movements, (7) biophysical profile score ≤6, (8) oligohydramnios, and (9) refusal to give informed consent.

### Intervention

First, gestational age (by fetal crown-rump length measurement at 11+0 to 13+6 weeks+days) [44] and EFW ≤10th percentile will be confirmed [45-49]. After giving their written informed consent, trial participants will be randomized into 2 groups: intervention and control.

In the intervention group, the sFlt-1/PlGF ratio will be revealed to investigators so they can act according to the results. When the sFlt-1/PlGF ratio is ≥38, the fetus will be classified as FGR. The remaining cases will be classified as SGA. In the intervention group, the UA PI, MCA PI, CPR, and UtA PI percentiles will be concealed to obstetricians in order to avoid any influence that this information might have on their interpretation of fetal movements or CTG.

In the control group, the sFlt-1/PlGF ratio will be concealed to investigators and the standard Doppler-based approach will be used for fetal monitoring [1]. Thus, fetuses with an EFW <3rd percentile or ≤10th percentile accompanied by an abnormal feto-maternal Doppler (UA PI >95th percentile, MCA PI <5th percentile, CPR <5th percentile, UtA PI >95th percentile, or a combination of these markers) [50-53] will be classified as FGR. The remaining cases will be classified as SGA.

In both groups, when a fetus is classified as FGR, immediate (within 24 hours) elective delivery at ≥37 weeks of gestation will be recommended; when a fetus is classified as SGA, elective delivery will be delayed until 40 weeks of gestation. From randomization to delivery, all SGA pregnancies in both groups will receive weekly follow-ups consisting of fetal ultrasound (including fetal growth, amniotic fluid deepest vertical pocket, fetal movements, and feto-maternal Doppler), conventional CTG, and measurement of the sFlt-1/PlGF ratio (which will be concealed or revealed depending on the allocated group).

**Figure 1:**
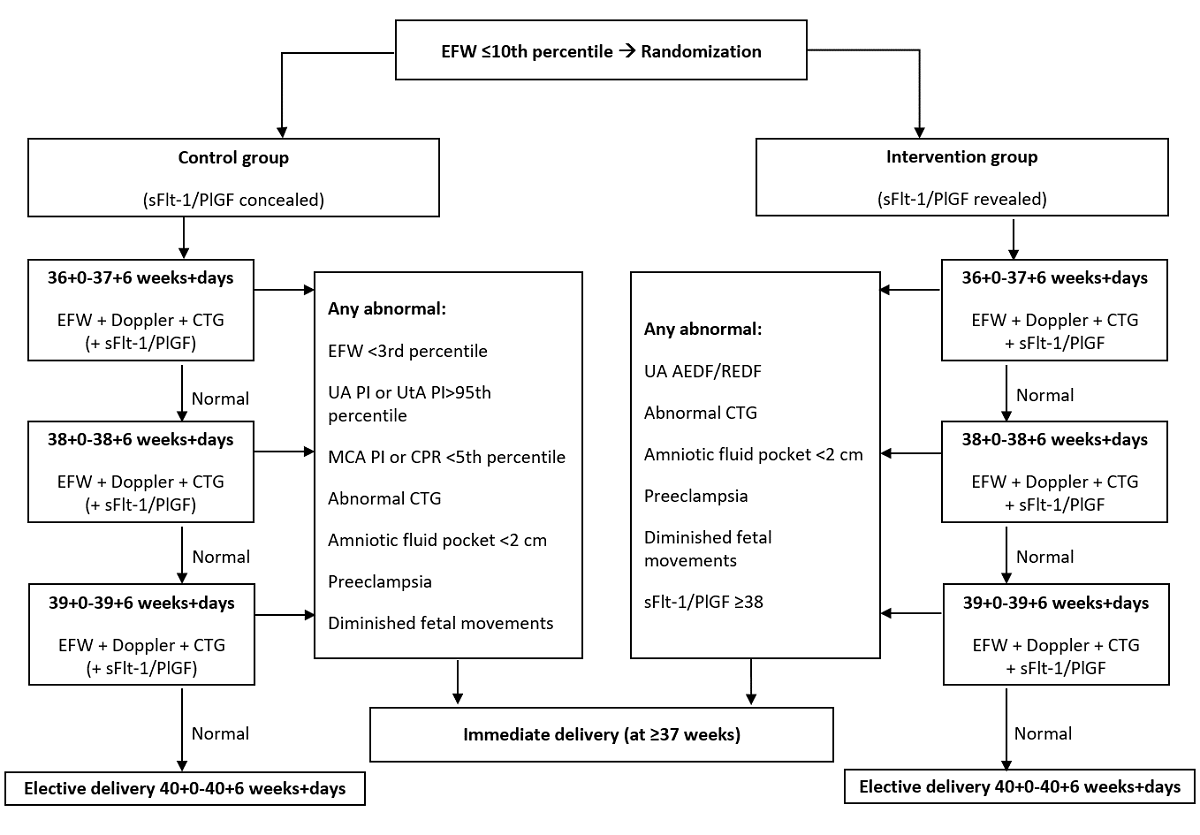
Flow chart of study intervention and management. AEDF: absent end-diastolic flow; CPR: cerebroplacental ratio; CTG: cardiotocography; EFW: estimated fetal weight; MCA: middle cerebral artery; PI: pulsatility index; PlGF: placental growth factor; REDF: reversed end-diastolic flow; sFlt-1: soluble fms-like tyrosine kinase-1; UA: umbilical artery.

In both groups, if at any time after enrollment any of the following is present, immediate (within 24 hours) delivery will be recommended: UA with absent or reversed end-diastolic flow, nonreassuring CTG, PE, diminished fetal movements, biophysical profile score ≤6, or oligohydramnios (largest vertical pocket <2 cm). The flow chart in Figure 1 illustrates the management of participants from consent through follow-up.

According to recommendations of the National Institute for Health and Care Excellence (NICE) [54], labor will be induced in pregnancies with a Bishop score [55] ≤6 by promoting cervical ripening with vaginal administration of dinoprostone or misoprostol or with a cervical balloon, as per each site’s usual protocols (Table 1).

In pregnancies with a Bishop score >6, labor will be induced by amniotomy, intravenous oxytocin infusion, or both in all participating sites. Indications for elective cesarean delivery will be as follows: at least 2 previous cesarean deliveries, UA with absent or reversed end-diastolic flow, nonreassuring CTG, abnormal fetal presentation (breech or transverse lie position), placental abruption, PE with severe features requiring immediate delivery (pulmonary edema, serum creatinine >1.1 mg/dL, oliguria [≤500 ml in 24 h or <20 ml/h], persistent hypertension despite appropriate antihypertensive therapy, persistent cerebral or visual disturbances, or eclampsia), and participants refusing induction of labor. Other less frequent indications may occur and will be classified as “other.” Indications for intrapartum cesarean delivery will be as follows: prolonged labor, failed induction of labor, nonreassuring CTG, placental abruption, and PE with severe features requiring immediate delivery. Other less frequent indications may occur and will be classified as “other.”

Prolonged labor will be defined according to the NICE guidelines for intrapartum care of healthy women and babies [56]. According to these guidelines, a delay in the first stage of labor is suspected if cervical dilatation is <2 cm after 4 hours. After 2 hours, delay will be confirmed if progress is <1 cm, and oxytocin will be offered. Prolonged labor will be confirmed if dilatation has increased <2 cm after 4 hours of oxytocin infusion. The maximum duration of cervical ripening treatment will vary depending on the method, with 12 hours for the cervical balloon, 16 hours for misoprostol, and 24 hours for dinoprostone. Failed induction of labor will be defined as not entering the active phase of labor after cervical ripening along with 6 to 8 hours of oxytocin infusion.

**Table 1:** Cervical ripening mechanisms used at each participating site. For labor induction, classification of fetuses as being small for gestational age or having fetal growth restriction will be based on the Doppler criteria, as in the control group [1]. Dinoprostone (Propess; Ferring Pharmaceuticals Ltd) was administered at a 10-mg dose with a vaginal delivery system. Misoprostol (Misofar; Exeltis Healthcare SL) was administered at a 25-µg dose with a vaginal tablet. Cervical balloons used a double-balloon catheter plus stylet (Cook Medical).

Hospital	Fetal growth restriction	Small for gestational age
Vall d’Hebron	Dinoprostone	Dinoprostone
Torrejón	Cervical balloon	Dinoprostone
Joan XXIII	Misoprostol	Misoprostol
Alicante	Dinoprostone	Dinoprostone
Arrixaca	Dinoprostone	Dinoprostone
Parc Taulí	Dinoprostone	Dinoprostone
Cabueñes	Dinoprostone	Dinoprostone
Germans Trias	Dinoprostone	Dinoprostone
Son Llàtzer	Dinoprostone	Dinoprostone
Lozano Blesa	Cervical balloon	Dinoprostone
Althaia	Dinoprostone	Dinoprostone
A Coruña	Dinoprostone	Dinoprostone
Elche	Cervical balloon	Dinoprostone
Valme	Cervical balloon	Cervical balloon
Hospital Terrassa	Dinoprostone	Misoprostol
Mutua Terrassa	Dinoprostone	Misoprostol
Getafe	Cervical balloon	Cervical balloon
Puerta del Mar	Cervical balloon	Misoprostol
Josep Trueta	Misoprostol	Misoprostol
Candelaria	Dinoprostone	Dinoprostone

### Predictive Variables

Predictive variables include maternal sFlt1 and PlGF plasma levels (pg/ml), fetal EFW, results of a Doppler assessment (UA PI, MCA PI, CPR, and UtA PI percentiles), amniotic fluid vertical pocket, fetal movement and biophysical profile score, and conventional CTG interpretation. PlGF and sFlt-1 levels will be measured using the automated Elecsys electrochemiluminescence immunoassay platform (Cobas Analyzers; Roche Diagnostics).

Nonreassuring CTG before and during labor will be defined as sinusoidal fetal heart rate tracing or absent fetal heart rate variability accompanied by recurrent late decelerations, recurrent variable decelerations, or bradycardia [57].

In all settings, EFW will be calculated using the Hadlock formula [49]. EFW percentiles will be calculated using the reference charts of each site’s protocol. Fetuses with an EFW ≤10th percentile will be classified as small [45-48]. Doppler assessments will be performed following the International Society of Ultrasound in Obstetrics and Gynecology Practice Guidelines [58]. All participating sites will use the same reference values for calculating UtA PI percentiles [51]. Doppler percentiles for UA PI, MCA PI, and CPR will be calculated according to gestational age using the charts of each site’s protocol (Table 2). Gestational age will be determined by fetal crown-rump length measurement at 11+0 to 13+6 weeks+days of gestation [44] or in vitro fertilization date.

**Table 2:** Reference charts at each participating site.

Hospital	Estimated fetal weight percentile	Umbilical artery pulsatility index percentile	Middle cerebral artery pulsatility index percentile	Cerebroplacental ratio percentile
Vall d’Hebron	Mikolajczyk RT et al [47]	Ciobanu A et al [50]	Ciobanu A et al [50]	Ciobanu A et al [50]
Torrejón	Marsál K et al [48]	Arduini D and Rizzo G [52]	Baschat AA and Gembruch U [53]	Baschat AA and Gembruch U [53]
Joan XXIII	Hadlock FP et al [49]	Arduini D and Rizzo G [52]	Baschat AA and Gembruch U [53]	Baschat AA and Gembruch U [53]
Alicante	Mikolajczyk et al [47]	Ciobanu A et al [50]	Ciobanu A et al [50]	Ciobanu A et al [50]
Arrixaca	Figueras F et al [45]	Arduini D and Rizzo G [52]	Baschat AA and Gembruch U [53]	Baschat AA and Gembruch U [53]
Parc Taulí	Hadlock FP et al [49]	Arduini D and Rizzo G [52]	Baschat AA and Gembruch U [53]	Baschat AA and Gembruch U [53]
Cabueñes	Hadlock FP et al [49]	Arduini D and Rizzo G [52]	Baschat AA and Gembruch U [53]	Baschat AA and Gembruch U [53]
Germans Trias	Hadlock FP et al [49]	Arduini D and Rizzo G [52]	Baschat AA and Gembruch U [53]	Baschat AA and Gembruch U [53]
Son Llàtzer	Figueras F et al [45]	Arduini D and Rizzo G [52]	Baschat AA and Gembruch U [53]	Baschat AA and Gembruch U [53]
Lozano Blesa	Figueras F et al [45]	Arduini D and Rizzo G [52]	Baschat AA and Gembruch U [53]	Baschat AA and Gembruch U [53]
Althaia	Figueras F et al [45]	Arduini D and Rizzo G [52]	Baschat AA and Gembruch U [53]	Baschat AA and Gembruch U [53]
A Coruña	Figueras F et al [45]	Arduini D and Rizzo G [52]	Baschat AA and Gembruch U [53]	Baschat AA and Gembruch U [53]
Elche	Papageorghiou AT et al [46]	Ciobanu A et al [50]	Ciobanu A et al [50]	Ciobanu A et al [50]
Valme	Figueras F et al [45]	Arduini D and Rizzo G [52]	Baschat AA and Gembruch U [53]	Baschat AA and Gembruch U [53]
Hospital Terrassa	Figueras F et al [45]	Arduini D and Rizzo G [52]	Baschat AA and Gembruch U [53]	Baschat AA and Gembruch U [53]
Mutua Terrassa	Hadlock FP et al [49]	Arduini D and Rizzo G [52]	Baschat AA and Gembruch U [53]	Baschat AA and Gembruch U [53]
Getafe	Figueras F et al [45]	Arduini D and Rizzo G [52]	Baschat AA and Gembruch U [53]	Baschat AA and Gembruch U [53]
Puerta del Mar	Mikolajczyk et al [47]	Ciobanu A et al [50]	Ciobanu A et al [50]	Ciobanu A et al [50]
Josep Trueta	Mikolajczyk et al [47]	Ciobanu A et al [50]	Ciobanu A et al [50]	Ciobanu A et al [50]
Candelaria	Figueras F et al [45]	Arduini D and Rizzo G [52]	Baschat AA and Gembruch U [53]	Baschat AA and Gembruch U [53]

Amniotic fluid volume will be determined measuring the deepest vertical pocket and oligohydramnios will be considered when depth is <2 cm [59]. Depending on each site’s protocol, fetal movement will be assessed subjectively or based on biophysical profile score, as described by Manning [60]. PE will be defined as new-onset high blood pressure (systolic blood pressure ≥140 mm Hg or diastolic blood pressure ≥90 mm Hg), worsening of previous high blood pressure in addition to new-onset proteinuria (≥300 mg protein in a 24-hour urine collection, protein/creatinine ≥0.3, or a dipstick reading of 1+), worsening of previous proteinuria, or according to at least one of the following signs and symptoms: cerebral or visual symptoms, raised liver enzymes, low platelet count, renal insufficiency, and pulmonary edema. PE with severe features will be defined as either systolic blood pressure ≥160 mm Hg or diastolic blood pressure ≥110 mm Hg, or PE with any of the following signs and symptoms: cerebral or visual symptoms, raised liver enzymes, low platelet count, renal insufficiency, and pulmonary edema [61].

### Outcomes

#### Primary Outcome

The primary outcome is the prevalence of cesarean delivery due to nonreassuring fetal status or the prevalence of neonatal acidosis. Neonatal acidosis will be defined as a UA pH <7.15 and a base excess greater than –12 mEq/L.

#### Secondary Outcomes

Composite adverse perinatal outcome will be defined as the presence of at least one of the following: fetal death, Apgar score <7 at 5 minutes, UA pH <7.05, admission to the NICU or a transitional care unit within 48 hours, birthweight <2000 grams, maternal admission to the obstetric intensive care unit within 48 hours (before or after delivery), and PE.

Composite adverse neonatal outcomes will be defined as the presence of at least one of the following: respiratory distress syndrome (respiratory rate >60 or <30 breaths/min, grunting on expiration, chest indrawing, central cyanosis, apnea, or the need for surfactant therapy in the neonatal period) [62], transient tachypnea, required ventilatory support, grade III or IV intraventricular hemorrhage, neonatal sepsis, hypoglycemia, necrotizing enterocolitis, neonatal jaundice (treated with phototherapy), neonatal seizures, pneumonia, meningitis, and neonatal death.

Other secondary outcomes will include the following: rates of elective delivery before 38, 39, and 40 weeks of gestation; rates of deliveries (elective and spontaneous) before 38, 39, and 40 weeks of gestation; rate of birthweight <2500 grams; rate of UA pH <7.10; rate of elective cesarean delivery; rate of cesarean delivery due to failed labor induction; rate of emergency operative vaginal delivery; and rate of placental-related complications, such as placental abruption, pregnancy hypertension, severe PE, eclampsia, stroke, maternal death, and postpartum hemorrhage.

### Statistical Analysis

Statistical analysis will be performed based on the intention-to-treat approach, considering all randomized women. A sensitivity analysis will be carried out to take into account the effect of withdrawal of consent and loss to follow-up. Outcomes and covariates will be imputed by multiple imputation chain equations. Patients deemed ineligible after randomization (eg, due to identification of congenital defects or EFW >10th percentile) will be excluded in the per-protocol analysis.

Univariate descriptive analysis will be used for the study variables. We will assess differences between the groups for the primary and secondary outcomes, calculating differences in the incidence and relative risks with their respective 95% CIs. Type I errors will be set at *P*<.05. The statistical software packages R and R Studio (R Foundation) will be used for statistical analyses. An interim analysis will be performed by an independent statistician once 50% of the sample size has been recruited. This analysis will ascertain the safety of the new approach with the O’Brien-Fleming boundary [63]. As FGR pregnancies have a higher risk of stillbirth and other adverse outcomes compared to SGA pregnancies, women with SGA fetuses will probably be more willing to participate. Enrollment of a greater proportion of SGA pregnancies might hinder identification of differences between groups. For this reason, a subgroup analysis will be performed for FGR and SGA pregnancies according to the Doppler classification at enrollment. Categorical variables will be reported as frequencies, normally distributed continuous variables will be reported as means and standard deviations, and continuous variables that do not follow a normal distribution will be reported as medians and interquartile ranges. The Fisher exact test or chi-square test, as appropriate, will be used to assess differences in categorical variables between groups. The Student *t* test (2-tailed) or Mann-Whitney *U* test, as appropriate, will be used for continuous variables.

During the design stage of the trial, no financial support was available. Nevertheless, if this trial receives a specific grant from a funding agency, monitoring by the Academic Research Organization of the Vall d’Hebron Research Institute will be contracted.

### Sample Size

A management protocol based on EFW and Doppler assessment has shown a prevalence of adverse perinatal outcomes of 26%, meaning that there is a prevalence of 74% of pregnancies with no complications [25]. The estimated rate of pregnancies with no complications in the intervention group has been set at 74%, with a lower limit of 65.5% (a maximum achievable difference of 8.5%). Based on these considerations and an estimated dropout rate of 3%, the sample size needed for a noninferiority design with a power of 80% and a significance level of 5% is 1030 participants, that is, 515 in each group. Noninferiority will be demonstrated if the lower limit of the 95% CI of the difference between incidences of pregnancies without neonatal acidosis is less than –8.5%. If the dropout rate is greater than 3%, the number of participants will be increased so as to achieve 1000 participants with complete data for the primary outcome.

### Randomization, Masking, and Data Collection

Participants will be randomly assigned to the intervention or control group in a 1:1 ratio using variable-size block randomization. The randomization sequence will be centralized and generated by the web-based system Sealed Envelope (Sealed Envelope Ltd) and will be concealed to investigators. Owing to the nature of the intervention, it will not be possible to conceal the study group to the participants, investigators, or outcome assessors.

A RedCap (Research Electronic Data Capture; Vanderbilt University) electronic database has been specifically designed for this study [64]. The electronic database has a randomization module that will allow allocation of participants to the study groups. Data will be entered prospectively during the study. Access to this database will be restricted to the investigators involved in each participating site.

### Ethics Approval

The current version (version 3.0) of the study protocol was approved by the Vall d’Hebron Ethics Committee (PR[AMI]527/2019) on February 18, 2020. Subsequent approval by individual ethical committees has been granted. Written informed consent will be obtained from all participants before randomization.

## Results

The first patient was recruited on September 28, 2020, and at the time of submitting this manuscript, the study was in the recruitment and data collection phase. The study results are expected to be published in peer-reviewed journals and disseminated at international conferences in early 2023. No funding has been obtained for this trial.

## Discussion

Newborns under 39 weeks have poorer perinatal outcomes than full-term newborns [10]. After classification with EFW and Doppler, more than 60% of small fetuses are delivered at 37 to 38 weeks of gestation [8]. However, classification with AF seems to have a lower false-positive rate [36]. In this trial, we aim to assess whether the classification of small fetuses as FGR or SGA based on AF is not inferior to the standard clinical approach (EFW and Doppler percentiles) for the identification of fetuses at a higher risk of adverse perinatal outcomes (neonatal acidosis and cesarean section due to nonreassuring CTG). This is the first trial that includes term pregnancies with an EFW below the 10th percentile and is designed to compare perinatal outcomes with a management protocol based on the sFlt-1/PlGF ratio and the standard management protocol, based on feto-maternal Doppler assessment. The main strength of this study is the comparison of 2 randomized groups and the large size of the study population. A pragmatic and multicenter design will evaluate the effectiveness of both interventions in the conditions of real-life routine practice, which will allow extrapolating the results to other settings. On the other hand, the sample size will not allow assessment of the effect of the management protocol on the incidence of rare adverse outcomes, such as stillbirth, placental abruption, or eclampsia. All pregnant women with fetuses having an EFW ≤10th percentile at 36+0 to 37+6 weeks+days of gestation will be invited to participate; however, since FGR pregnancies are at a higher risk of stillbirth and other adverse outcomes, women with FGR pregnancies might be more reluctant to participate than women with SGA, which could introduce a selection bias.

The AF-based protocol may reduce the number of pregnancies classified as FGR without worsening perinatal outcomes, improve patients’ medical attention perception, reduce the rate of cesarean deliveries, and reduce the rate of placental complications, such as PE, placental abruption, or eclampsia. Moreover, the rate of early-term neonates may be reduced, improving neonatal outcomes and long-term morbidity.

## Abbreviations

AF: angiogenic factors
CPR: cerebroplacental ratio
CTG: cardiotocography
EFW: estimated fetal weight
FGR: fetal growth restriction
MCA: middle cerebral artery
NICE: National Institute for Health and Care Excellence
NICU: neonatal intensive care unit
PE: preeclampsia
PI: pulsatility index
PlGF: placental growth factor
sFlt-1: soluble fms-like tyrosine kinase-1
SGA: small for gestational age
SPIRIT: Standard Protocol Items: Recommendations for Interventional Trials
UA: umbilical artery
UtA: uterine artery

## Data Availability

The data that support the findings of this study are available from the corresponding author, MM, upon reasonable request.

## References

[1] Figueras F, Gratacós Eduard (2014). Update on the diagnosis and classification of fetal growth restriction and proposal of a stage-based management protocol. Fetal Diagn Ther.

[2] McCowan LM, Figueras F, Anderson NH (2018). Evidence-based national guidelines for the management of suspected fetal growth restriction: comparison, consensus, and controversy. Am J Obstet Gynecol.

[3] Lees CC, Marlow N, van Wassenaer-Leemhuis Aleid, Arabin B, Bilardo CM, Brezinka C, Calvert S, Derks JB, Diemert A, Duvekot JJ, Ferrazzi E, Frusca T, Ganzevoort W, Hecher K, Martinelli P, Ostermayer E, Papageorghiou AT, Schlembach D, Schneider KTM, Thilaganathan B, Todros T, Valcamonico A, Visser GHA, Wolf H, TRUFFLE study group (2015). 2 year neurodevelopmental and intermediate perinatal outcomes in infants with very preterm fetal growth restriction (TRUFFLE): a randomised trial. Lancet.

[4] Meler E, Mazarico E, Eixarch E, Gonzalez A, Peguero A, Martinez J, Boada D, Vellvé K, Gomez-Roig MD, Gratacós E, Figueras F (2021). Ten-year experience of protocol-based management of small-for-gestational-age fetuses: perinatal outcome in late-pregnancy cases diagnosed after 32 weeks. Ultrasound Obstet Gynecol.

[5] Savchev S, Figueras F, Sanz-Cortes M, Cruz-Lemini M, Triunfo S, Botet F, Gratacos E (2014). Evaluation of an optimal gestational age cut-off for the definition of early- and late-onset fetal growth restriction. Fetal Diagn Ther.

[6] Baschat AA (2018). Planning management and delivery of the growth-restricted fetus. Best Pract Res Clin Obstet Gynaecol.

[7] Bond DM, Gordon A, Hyett J, de Vries Bradley, Carberry Angela E, Morris Jonathan (2015). Planned early delivery versus expectant management of the term suspected compromised baby for improving outcomes. Cochrane Database Syst Rev.

[8] Figueras F, Gratacós E (2017). An integrated approach to fetal growth restriction. Best Pract Res Clin Obstet Gynaecol.

[9] Gordijn SJ, Beune IM, Thilaganathan B, Papageorghiou A, Baschat AA, Baker PN, Silver RM, Wynia K, Ganzevoort W (2016). Consensus definition of fetal growth restriction: a Delphi procedure. Ultrasound Obstet Gynecol.

[10] Sengupta S, Carrion V, Shelton J, Wynn RJ, Ryan RM, Singhal K, Lakshminrusimha S (2013). Adverse neonatal outcomes associated with early-term birth. JAMA Pediatr.

[11] Paz Levy D, Sheiner E, Wainstock T, Sergienko R, Landau D, Walfisch A (2017). Evidence that children born at early term (37-38 6/7 weeks) are at increased risk for diabetes and obesity-related disorders. Am J Obstet Gynecol.

[12] Walfisch A, Beharier O, Wainstock T, Sergienko R, Landau D, Sheiner E (2017). Early-term deliveries as an independent risk factor for long-term respiratory morbidity of the offspring. Pediatr Pulmonol.

[13] Altshuler G, Russell P, Ermocilla R (1975). The placental pathology of small-for-gestational age infants. Am J Obstet Gynecol.

[14] Zur RL, Kingdom JC, Parks WT, Hobson SR (2020). The placental basis of fetal growth restriction. Obstet Gynecol Clin North Am.

[15] Paules C, Youssef L, Rovira C, Crovetto F, Nadal A, Peguero A, Figueras F, Eixarch E, Crispi F, Miranda J, Gratacós E (2019). Distinctive patterns of placental lesions in pre-eclampsia vs small-for-gestational age and their association with fetoplacental Doppler. Ultrasound Obstet Gynecol.

[16] Roberts DJ, Post MD (2008). The placenta in pre-eclampsia and intrauterine growth restriction. J Clin Pathol.

[17] Ganer Herman H, Barber E, Gasnier R, Gindes L, Bar J, Schreiber L, Kovo M (2018). Placental pathology and neonatal outcome in small for gestational age pregnancies with and without abnormal umbilical artery Doppler flow. Eur J Obstet Gynecol Reprod Biol.

[18] Shinar S, Tigert M, Agrawal S, Parks WA, Kingdom John C (2021). Placental growth factor as a diagnostic tool for placental mediated fetal growth restriction. Pregnancy Hypertens.

[19] Triunfo S, Lobmaier S, Parra-Saavedra M, Crovetto F, Peguero A, Nadal A, Gratacos E, Figueras F (2014). Angiogenic factors at diagnosis of late-onset small-for-gestational age and histological placental underperfusion. Placenta.

[20] Garcia-Manau Pablo, Mendoza M, Bonacina E, Garrido-Gimenez Carmen, Fernandez-Oliva Antoni, Zanini J, Catalan M, Tur H, Serrano B, Carreras E (2021). Soluble fms-like tyrosine kinase to placental growth factor ratio in different stages of early-onset fetal growth restriction and small for gestational age. Acta Obstet Gynecol Scand.

[21] Savchev S, Figueras F, Cruz-Martinez R, Illa M, Botet F, Gratacos E (2012). Estimated weight centile as a predictor of perinatal outcome in small-for-gestational-age pregnancies with normal fetal and maternal Doppler indices. Ultrasound Obstet Gynecol.

[22] NIETO A (2009). Neonatal morbidity associated with disproportionate intrauterine growth retardation at term. Journal of Obstetrics and Gynaecology.

[23] Pilliod RA, Cheng YW, Snowden JM, Doss AE, Caughey AB (2012). The risk of intrauterine fetal death in the small-for-gestational-age fetus. American Journal of Obstetrics and Gynecology.

[24] Boers KE, Vijgen SMC, Bijlenga D, van der Post JAM, Bekedam DJ, Kwee A, van der Salm PCM, van Pampus MG, Spaanderman MEA, de Boer K, Duvekot JJ, Bremer HA, Hasaart THM, Delemarre FMC, Bloemenkamp KWM, van Meir CA, Willekes C, Wijnen EJ, Rijken M, le Cessie S, Roumen FJME, Thornton JG, van Lith JMM, Mol BWJ, Scherjon SA (2010). Induction versus expectant monitoring for intrauterine growth restriction at term: randomised equivalence trial (DIGITAT). BMJ.

[25] Figueras F, Savchev S, Triunfo S, Crovetto F, Gratacos E (2015). An integrated model with classification criteria to predict small-for-gestational-age fetuses at risk of adverse perinatal outcome. Ultrasound Obstet Gynecol.

[26] Cruz-Martinez R, Savchev S, Cruz-Lemini M, Mendez A, Gratacos E, Figueras F (2015). Clinical utility of third-trimester uterine artery Doppler in the prediction of brain hemodynamic deterioration and adverse perinatal outcome in small-for-gestational-age fetuses. Ultrasound Obstet Gynecol.

[27] DeVore GR (2015). The importance of the cerebroplacental ratio in the evaluation of fetal well-being in SGA and AGA fetuses. American Journal of Obstetrics and Gynecology.

[28] Martinez-Portilla RJ, Caradeux J, Meler E, Lip-Sosa DL, Sotiriadis A, Figueras F (2020). Third-trimester uterine artery Doppler for prediction of adverse outcome in late small-for-gestational-age fetuses: systematic review and meta-analysis. Ultrasound Obstet Gynecol.

[29] Mureșan D, Rotar IC, Stamatian F (2016). The usefulness of fetal Doppler evaluation in early versus late onset intrauterine growth restriction. Review of the literature. Med Ultrason.

[30] Doctor BA, O'Riordan M A, Kirchner H, Shah D, Hack M (2001). Perinatal correlates and neonatal outcomes of small for gestational age infants born at term gestation. Am J Obstet Gynecol.

[31] Rizzo G, Mappa I, Bitsadze V, Słodki M, Khizroeva J, Makatsariya A, D'Antonio F (2020). Role of Doppler ultrasound at time of diagnosis of late-onset fetal growth restriction in predicting adverse perinatal outcome: prospective cohort study. Ultrasound Obstet Gynecol.

[32] Khalil A, Thilaganathan B (2017). Role of uteroplacental and fetal Doppler in identifying fetal growth restriction at term. Best Pract Res Clin Obstet Gynaecol.

[33] Herraiz I, Simón E, Gómez-Arriaga P I, Quezada MS, García-Burguillo A, López-Jiménez E A, Galindo A (2018). Clinical implementation of the sFlt-1/PlGF ratio to identify preeclampsia and fetal growth restriction: A prospective cohort study. Pregnancy Hypertens.

[34] Herraiz I, Quezada MS, Rodriguez-Calvo J, Gómez-Montes E, Villalaín C, Galindo A (2018). Longitudinal change of sFlt-1/PlGF ratio in singleton pregnancy with early-onset fetal growth restriction. Ultrasound Obstet Gynecol.

[35] Kwiatkowski S, Bednarek-Jędrzejek M, Ksel J, Tousty P, Kwiatkowska E, Cymbaluk A, Rzepka R, Chudecka-Głaz A, Dołęgowska B, Torbè Andrzej (2018). sFlt-1/PlGF and Doppler ultrasound parameters in SGA pregnancies with confirmed neonatal birth weight below 10th percentile. Pregnancy Hypertens.

[36] Gaccioli F, Sovio U, Cook E, Hund M, Charnock-Jones Ds, Smith Gcs (2018). Screening for fetal growth restriction using ultrasound and the sFLT1/PlGF ratio in nulliparous women: a prospective cohort study. Lancet Child Adolesc Health.

[37] Lees CC, Stampalija T, Baschat A, da Silva Costa F, Ferrazzi E, Figueras F, Hecher K, Kingdom J, Poon LC, Salomon L J, Unterscheider J (2020). ISUOG Practice Guidelines: diagnosis and management of small-for-gestational-age fetus and fetal growth restriction. Ultrasound Obstet Gynecol.

[38] Melamed N, Baschat A, Yinon Y, Athanasiadis A, Mecacci F, Figueras F, Berghella V, Nazareth A, Tahlak M, McIntyre HD, Da Silva Costa Fabrício, Kihara AB, Hadar E, McAuliffe F, Hanson M, Ma RC, Gooden R, Sheiner E, Kapur A, Divakar H, Ayres-de-Campos D, Hiersch L, Poon LC, Kingdom J, Romero Roberto, Hod Moshe (2021). FIGO (international Federation of Gynecology and obstetrics) initiative on fetal growth: best practice advice for screening, diagnosis, and management of fetal growth restriction. Int J Gynaecol Obstet.

[39] Zeisler H, Llurba E, Chantraine F, Vatish M, Staff AC, Sennström Maria, Olovsson M, Brennecke SP, Stepan H, Allegranza D, Dilba P, Schoedl M, Hund M, Verlohren S (2016). Predictive value of the sFlt-1:PlGF ratio in women with suspected preeclampsia. N Engl J Med.

[40] Zeisler H, Llurba E, Chantraine FJ, Vatish M, Staff AC, Sennström M, Olovsson M, Brennecke SP, Stepan H, Allegranza D, Schoedl M, Grill S, Hund M, Verlohren S (2019). Soluble fms-like tyrosine kinase-1 to placental growth factor ratio: ruling out pre-eclampsia for up to 4 weeks and value of retesting. Ultrasound Obstet Gynecol.

[41] Levine RJ, Maynard SE, Qian C, Lim K, England LJ, Yu KF, Schisterman EF, Thadhani R, Sachs BP, Epstein FH, Sibai BM, Sukhatme VP, Karumanchi SA (2004). Circulating angiogenic factors and the risk of preeclampsia. N Engl J Med.

[42] Signore C, Mills JL, Qian C, Yu K, Lam C, Epstein FH, Karumanchi SA, Levine RJ (2006). Circulating angiogenic factors and placental abruption. Obstet Gynecol.

[43] Chan A, Tetzlaff JM, Altman DG, Laupacis A, Gøtzsche Peter C, Krleža-Jerić Karmela, Hróbjartsson Asbjørn, Mann H, Dickersin K, Berlin JA, Doré Caroline J, Parulekar WR, Summerskill WSM, Groves T, Schulz KF, Sox HC, Rockhold FW, Rennie Drummond, Moher David (2013). SPIRIT 2013 statement: defining standard protocol items for clinical trials. Ann Intern Med.

[44] Robinson HP, Fleming JE (1975). A critical evaluation of sonar "crown-rump length" measurements. Br J Obstet Gynaecol.

[45] Figueras F, Meler E, Iraola A, Eixarch E, Coll O, Figueras J, Francis A, Gratacos E, Gardosi J (2008). Customized birthweight standards for a Spanish population. Eur J Obstet Gynecol Reprod Biol.

[46] Papageorghiou AT, Ohuma EO, Altman DG, Todros T, Cheikh Ismail Leila, Lambert A, Jaffer YA, Bertino E, Gravett MG, Purwar M, Noble JA, Pang R, Victora CG, Barros FC, Carvalho M, Salomon LJ, Bhutta ZA, Kennedy SH, Villar J, International Fetal and Newborn Growth Consortium for the 21st Century (INTERGROWTH-21st) (2014). International standards for fetal growth based on serial ultrasound measurements: the Fetal Growth Longitudinal Study of the INTERGROWTH-21st Project. Lancet.

[47] Mikolajczyk RT, Zhang J, Betran AP, Souza JP, Mori R, Gülmezoglu A Metin, Merialdi M (2011). A global reference for fetal-weight and birthweight percentiles. Lancet.

[48] Marsál K, Persson P, Larsen T, Lilja H, Selbing A, Sultan B (1996). Intrauterine growth curves based on ultrasonically estimated foetal weights. Acta Paediatr.

[49] Hadlock FP, Harrist R, Sharman RS, Deter RL, Park SK (1985). Estimation of fetal weight with the use of head, body, and femur measurements--a prospective study. Am J Obstet Gynecol.

[50] Ciobanu A, Wright A, Syngelaki A, Wright D, Akolekar R, Nicolaides KH (2019). Fetal Medicine Foundation reference ranges for umbilical artery and middle cerebral artery pulsatility index and cerebroplacental ratio. Ultrasound Obstet Gynecol.

[51] Gómez O, Figueras F, Fernández S, Bennasar M, Martínez J M, Puerto B, Gratacós E (2008). Reference ranges for uterine artery mean pulsatility index at 11-41 weeks of gestation. Ultrasound Obstet Gynecol.

[52] Arduini D, Rizzo G (1990). Normal values of Pulsatility Index from fetal vessels: a cross-sectional study on 1556 healthy fetuses. J Perinat Med.

[53] Baschat AA, Gembruch U (2003). The cerebroplacental Doppler ratio revisited. Ultrasound Obstet Gynecol.

[54] Overview | Inducing labour | Guidance | NICE. National Institute for Health and Care Excellence.

[55] Bishop EH (1964). Pelvic scoring for elective induction. Obstet Gynecol.

[56] Overview | Intrapartum care for healthy women and babies | Guidance | NICE. National Institute for Health and Care Excellence.

[57] Macones GA, Hankins GDV, Spong CY, Hauth John, Moore Thomas (2008). The 2008 National Institute of Child Health and Human Development workshop report on electronic fetal monitoring: update on definitions, interpretation, and research guidelines. J Obstet Gynecol Neonatal Nurs.

[58] Bhide A, Acharya G, Baschat A, Bilardo CM, Brezinka C, Cafici D, Ebbing C, Hernandez-Andrade E, Kalache K, Kingdom J, Kiserud T, Kumar S, Lee W, Lees C, Leung KY, Malinger G, Mari G, Prefumo F, Sepulveda W, Trudinger B (2021). ISUOG Practice Guidelines (updated): use of Doppler velocimetry in obstetrics. Ultrasound Obstet Gynecol.

[59] Shrem G, Nagawkar SS, Hallak Mordechai, Walfisch Asnat (2016). Isolated oligohydramnios at term as an indication for labor induction: a systematic review and meta-analysis. Fetal Diagn Ther.

[60] Manning FA (1999). Fetal biophysical profile. Obstet Gynecol Clin North Am.

[61] (2013). Hypertension in pregnancy. Report of the American College of Obstetricians and Gynecologists’ Task Force on Hypertension in Pregnancy. Obstet Gynecol.

[62] Sweet LR, Keech C, Klein NP, Marshall HS, Tagbo BN, Quine D, Kaur P, Tikhonov I, Nisar MI, Kochhar S, Muñoz Flor M, Brighton Collaboration Respiratory Distress in the Neonate Working Group (2017). Vaccine.

[63] Kumar A, Chakraborty B (2016). Interim analysis: A rational approach of decision making in clinical trial. J Adv Pharm Technol Res.

[64] Vall d'Hebron Research Institute (VHIR) Redcap installation. Vanderbilt University.

